# Supplementary material for: Are carbon emissions trading and green financial instruments synergistic? -Comprehensive quantitative research based on content analysis
Source: PLoS One. 2024 Mar 7;19(3):e0298601. doi: 10.1371/journal.pone.0298601 (PMC10919601; doi:10.1371/journal.pone.0298601)
Supplement: S1 Data — (ZIP) [file pone.0298601.s001.zip › Data/地级市Z_F工作报告.zip!/╕ú╜¿/╚2├≈╩╨/└┤╫╘╣1⁄2╓┌║┼í╛┬φ┐╦╩2╛▌═°í┐_2009.docx]

各位代表：

　　现在，我代表三明市人民政府向大会报告政府工作，请予审议，并请市政协委员和其他列席人员提出意见。

　　一、2008年工作回顾

　　2008年，我市各级政府在省委、省政府和市委正确领导下，认真贯彻党的十七大、十七届三中全会精神，以邓小平理论和“三个代表”重要思想为指导，深入落实科学发展观，推进海峡西岸两个先行区建设，经过全市人民共同努力，经济社会保持又好又快发展态势。初步统计，全市生产总值671亿元，比上年增长14.7%；三次产业结构从上年的21.8:43.3:34.9调整为20.6:47.9:31.5；全社会固定资产投资508.4亿元，增长40%；地方级财政一般预算收入32.57亿元，增长18.5%；按可比口径实际利用外商直接投资2.3亿美元，增长21%；外贸出口6.75亿美元，下降16%,其中生产型企业出口增长51.2%；社会消费品零售总额182亿元，增长22.2%；城镇居民人均可支配收入16013元，实际增长7%；农民人均纯收入5835元，实际增长8.8%；居民消费价格指数105%;城镇登记失业率3.7%；人口自然增长率6‰。一些重要领域、重点项目取得突破：

　　———泉（州）三（明）高速公路三明段建成通车，高速公路通车里程368公里，居九个设区市第一；永（安）武（平）高速公路三明段实现路基单幅贯通，永（安）宁（化）高速公路开工建设；向（塘）莆（田）铁路全线开工建设。

　　———冶金及压延产业集群产值突破200亿元；厦工三重、双轮化机、三明机床等企业完成异地搬迁扩建。街面水电站建成投产。

　　———泰宁南方林业、三明华健生物2家企业分别在法国、美国上市，实现我市企业境外上市零的突破。全省首家由外资银行出资设立的福建永安汇丰村镇银行有限责任公司挂牌成立。

　　———“大尺寸矩形平面光学零件高精度磨床”项目列入国家“863”计划，这是该计划设立以来，我市单独承担的首个课题。

　　———在全国率先实行新型农村合作医疗补充医疗保险，患者人均得到赔付5836元；全面启动城镇居民医疗保险，进度全省最快；我市被评为全国无偿献血先进城市。

　　一年来，我们立足好中求快，壮大重点产业，经济运行质量提升。

　　继续集中4000万元资金扶持工业发展，规模以上工业增加值271亿元，增长25%；“4+1”产业集群产值增长30%，占规模以上工业产值的64%；净增规模以上工业企业234家，新增产值超亿元企业30家；33个工业园区（集中区）新增入园企业77家，新增产值56亿元。建筑业加快发展，产值62亿元，增长28.3%。

　　持续加强农业，农林牧渔业总产值220亿元，增长6%。超级稻种植面积全省第一，粮食总产量112.92万吨。新增1家国家级龙头企业、7家省级龙头企业，市级以上龙头企业实现产值93亿元。农业社会化服务体系不断健全，新增200家农民专业合作社。新增5个国家级农业标准示范区，5个项目通过GAP认证。促进和规范农村土地承包经营权流转，耕地流转面积达到38万亩。

　　加快服务业发展，三明农资物流配送中心、沙县小吃文化城等项目建成投入使用，海西金属材料制品市场、三明城市物流园区等项目加快推进，新华都、苏宁电器等知名商贸企业入驻市区。全市旅游发展总体规划通过专家评审，游客接待量725万人次，增长16.4%；旅游总收入25亿元，增长16.8%。房地产业有序发展，投资增长41.5%。

　　注重提升经济运行质量，规模以上工业企业经济效益综合指数205%，比上年提高20个百分点。创新体系不断完善，58个项目列入国家、省科技计划，180项专利获得国家授权，新增6家国家级高新技术企业；国家（三明）人造板及林化工产品质量监督检验中心项目建成，机械先进制造公共服务平台投入运行，“6·18”项目成果交易会签约合同项目数和投资额居全省首位。品牌提升取得突破，国家地理标志产品保护达到7项，数量居全省第一；新增62个有机食品、绿色食品、无公害农产品。我市企业参与制定1项国际标准，主导制定3项国家标准和1项行业标准，2家企业获批筹建国家标准化委员会工作组。

　　突出可持续发展，抓好节能减排，预计二氧化硫削减率为3%，化学需氧量削减率为0.3%。实施36个重点节能项目，淘汰落后水泥产能120万吨，新增新型干法水泥产能200万吨，单位生产总值能耗下降4.2%。实施89个减排项目，三明市区空气优良天数占总天数的92.9%，比上年提高9.1个百分点；完成110个乡镇水源保护区划定工作，建成泰宁县、沙县污水处理厂和将乐、清流、宁化县垃圾无害化处理场。自然保护区建设与管理加强，明溪县被命名为国家级生态示范区。

　　一年来，我们强化项目带动，加快基础设施建设，城乡实力增强。

　　扎实推进重点项目建设，55个省级重点项目预计完成投资90.5亿元，139个市级重点项目预计完成投资125.5亿元，其中50个重点项目建成投产或部分投产。投资亿元以上的在建项目达到110个，其中亿元以上的在建工业项目59个，完成投资41.2亿元，增长35.5%。

　　持续加快基础设施建设，建（宁）泰（宁）、三（明）厦（门）、永（安）漳（州）高速公路前期工作扎实推进，三明绕城、尤溪至莆田兴化湾、三明至明溪高速公路列入海西高速公路网规划；国省道“改善工程”完成89公里；

　　铁道部和省政府确定2010年南（平）三（明）龙（岩）、浦（城）建（宁）龙（岩）铁路开工，2011年长（汀）永（安）泉（州）铁路开工；沙溪河二期航道整治工程完成；三明综合交通枢纽完成规划编制。永安火电厂扩改项目1台机组动工建设，500千伏超高压电网“南联北接”工程全面推进，220千伏主干电网建设向清流、尤溪、大田、泰宁等县延伸，一批城市电网项目和新农村电气化工程进展顺利。

　　发展壮大区域经济，三明市区、永安、沙县“主轴”的带动能力提升，地区生产总值占全市的54.5%；尤溪、大田县所在的“东南一翼”和将乐、泰宁、建宁、宁化、明溪、清流县所在的“西北一翼”特色更加鲜明，地区生产总值分别增长15.7%和14%。市区经济持续发展，冶金、机械、生物医药等产业不断壮大，市区生产总值达到162亿元，占全市的24.3%，比上年提高0.9个百分点。贵溪洋大道建设稳步推进，徐碧大桥至陈大及翁墩立交、工业南路至泉三高速公路南互通通道动工。徐碧新城、下洋中区、台江新区开发有序推进，徐碧中央商务区动工，市科技会展中心投入使用，台江大桥进入主梁铺设阶段。投资1.6亿元改善市政和社区基础设施，更新市区公交车辆77辆，国家园林城市通过省级复查验收。

　　有序推进新农村建设，十件实事完成，30个示范村、200个推进村的工作进一步加强。投入11.3亿元，实施一批水利、烟田、农田等基础设施项目；55个乡镇、593个建制村通过省级农村家园清洁行动验收。11件老区办实事项目完成，沙县、将乐县被确认为中央苏区县。省、市级扶贫重点村争取资金1.2亿元，扶贫工作取得进展。

　　一年来，我们突出主动运作，积极应对困难，发展态势持续向好。

　　面对年初严重的冰冻灾害，我们把灾区群众的冷暖安危放在首位，迅速组织修复电力、通信、交通设施，及时安置、疏导滞留旅客和车辆，千方百计保畅通、保供应、保安全、保生产，确保灾区人民过上欢乐祥和的春节。特别是受灾严重的建宁县短时间内恢复生产生活秩序，经济社会实现持续较快发展。四川汶川特大地震发生后，我们全力支援抗震救灾，捐款数额达到7368万元，创历年单次救灾捐赠最高水平；对口支援彭州市红岩镇，2个援建项目已经完成，3个产业项目抓紧实施。

　　面对国际金融危机和宏观政策调整，我们主动作为，制定扶持中小企业加快发展的33条意见，出台支持中央属企业落地三明的10条举措，加强煤电油运调度，帮助企业克服困难。我们有序运作，有效调度财政资金，组织3次政银企对接活动，制定改善小企业金融服务的15条意见，出台加强农村金融服务的7条措施，深化与政策性金融机构合作，增加担保机构风险补偿金，全年调度财政资金7.05亿元，金融机构新增贷款65亿元，新增开发性金融合作贷款36.93亿元，全市24家担保机构为企业提供31.1亿元贷款担保，融资困难得到缓解。我们抢抓机遇，迅速落实扩大内需政策，截至12月中旬，省发改委批转下达我市新增中央投资计划89项，新增中央预算内投资2.08亿元。我们积极争取，在实现耕地占补平衡的同时，提供项目用地10066亩。

　　一年来，我们深化改革创新，扩大对外开放，经济主体日趋活跃。

　　深化各项改革，集体林权制度改革继续走在全国前列，生态公益林管护机制改革基本完成，林业小额贷款金额居全省第一。三农（集团）公司股权分置改革有序推进，企业改制上市工作取得突破。市级财政国库集中支付改革继续深化。第二批乡镇机构改革试点工作启动，事业单位人事制度改革取得成效，市级事业单位机构编制清理整顿工作基本完成。

　　扩大对外开放，成功举办第四届林博会，赴香港、北京、上海、广东佛山、泉州等地开展专场招商，参加“9·8”等招商活动，引进区外资金和外资72.88亿元，增长35.6%。支持生产型出口企业发展，3家企业入选省级出口名牌企业。加快建设海峡两岸（三明）现代林业合作实验区和清流台湾农民创业园，新批办12家台资企业。明港澳合作深化，与海外华侨及国际友城联系加强，海关、检验检疫、贸易促进等工作取得新成效，8件外商投诉的历年积案得到妥善解决。

　　加快发展非公有制经济，实现产值增长25%、税收增长24%，新增32个投资超亿元非公项目、10家产值超亿元的非公企业、40家纳税百万元以上非公企业。新组建4个商会，市区9个商会实施项目153个，完成投资73.36亿元。总部经济有序发展，引进2家营销总部或分支机构落户三明。

　　一年来，我们加强公共服务，推进平安建设，社会保持和谐稳定。

　　推动社会事业协调发展，义务教育学龄人口入学率继续保持全省较高水平，清流县通过“双高普九”省级验收，职业教育、高等教育、学前教育稳步发展。加强人才队伍建设，引入一批高层次人才和紧缺急需专业毕业生，引进国外智力项目21个，争取国家和省里引智资金居全省前列。沙县、梅列区被评为全国科普示范县（区），社会科学取得新成绩。公共卫生体系不断健全，重大疾病防控工作得到加强，医疗收费进一步规范。公共文化体系逐步完善，建立4个省级文化产业示范基地，大田板凳龙、将乐竹纸制作技艺入选第二批国家级非物质文化遗产保护名录。沙县、宁化县分别被评为全国、全省计划生育优质服务先进单位，人口与计生工作完成省下达的年度责任目标。全民健身运动深入开展，我市运动员张杰在亚洲举重锦标赛上打破世界记录，田径选手王静入选参加北京奥运会。统计工作服务水平提高，第二次全国农业普查顺利完成，物价、民族宗教、水文、气象、地震和地方志工作取得新进展，妇女、儿童、残疾人和未成年人工作加强。

　　推进民主法制和精神文明建设，完善村（居）民自治，基层民主逐步扩大。“五五”普法深入开展。群众性精神文明创建活动深入推进，“共建共享和谐三明”主题活动扎实开展，创建全国文明城市工作取得成效。双拥共建持续深化，民兵预备役、人民防空和国防后备力量建设继续加强。社会治安综合治理和“平安三明”建设取得明显成效，12个县（市、区）全部达到平安县（市、区）标准，社会治安防控体系日趋完善，群众对社会治安满意率达到96.8%。信访工作不断加强，一批信访突出问题和矛盾纠纷有效化解。突发公共事件应急管理体系不断健全，安全生产“隐患治理年”和“基层基础建设年”成效明显。

　　一年来，我们落实就业政策，解决民生问题，群众得到更多实惠。

　　加强就业服务，新增城镇就业2.3万人，农村劳动力转移培训3.7万人，实现转移就业4.55万人。健全社会保障体系，完成省政府下达的医疗、工伤和生育保险任务，企业退休人员人均月增加养老金118元，医改前退休人员的医保问题得到妥善解决。完善社会救助体系，城市低保人均月补助水平提高42元，从7月1日起农村低保人均月补助水平提高10元，农村五保户供养水平接近当地村民平均生活水平。老干部政策得到落实，老龄事业进一步发展，市老年活动中心动工，社会慈善、社会捐赠和群众互助公益活动取得实效。

　　办实事项目完成年度任务，教育“两免一补”惠及学生31万名；新建农村中小学寄宿生宿舍14.59万平方米，改造、修缮农村中小学危房14.82万平方米。26所乡镇卫生院、22个乡镇综合文化站和16所农村敬老院建设、改造任务完成，广播电视村村通工程提前两年半完成。新建经济适用住房26万平方米、廉租住房1.2万平方米;“造福工程”年度搬迁9150人；侨居造福工程基本完成。新建通建制村公路128公里，硬化路面通村率达99%以上；农村户用沼气完成1万口，“六千”水利工程完成年度目标；海西三明气象中心等项目进展顺利，防洪“预警到乡”工程建成，政策性农业保险试点工作扎实推进。食品药品安全专项整治深入开展，乳制品质量安全专项清查取得成效。发放库区后期扶持直补资金和投入项目补助资金1.16亿元。

　　各位代表，提升行政能力是做好政府工作的重要保障。一年来，围绕建设开明政府，自觉接受人大及其常委会的法律监督、工作监督和政协的民主监督，认真办理市人大代表建议222件、市政协提案494件，办结率均为100%；落实市人大常委会关于确保市区居民饮水安全议案的决议精神，东牙溪饮用水源保护得到加强，供水安全监管、水库安全管理、饮用水卫生监督落实到位，市区第二饮用水源建设前期工作展开。密切与各民主党派、工商联、无党派人士的联系与协商，支持工会、共青团、妇联等群团组织工作。实行政府信息公开，政府工作透明度提高。围绕建设服务政府，减少行政审批事项168项，建成市县乡联动的网上审批服务体系和电子监察系统，绩效管理加强，机关效能提高，发展环境改善；完善重大项目工作组运作模式，推进的65个重大项目进展明显。围绕建设法治政府，开展法律知识专题培训，出台重大事项决策程序规定，落实行政执法责任制，加强行政复议工作，依法行政水平得到提高。围绕建设廉洁政府，健全惩治和预防腐败体系，推进公共资源市场化配置，规范行政自由裁量权，深入开展纠风工作，接受群众和社会舆论监督，推进行政监察、审计监督，从严治政工作得到加强。

　　各位代表，过去一年的成绩来之不易，是在省委、省政府和市委正确领导下，在历届政府打下的良好基础上，经过全市上下奋力拼搏取得的。在此，我谨代表市人民政府，向辛勤工作在各条战线的全市人民，向给予政府大力支持与有效监督的人大代表和政协委员、各民主党派、工商联、各人民团体、无党派人士、离退休老同志、社会各界人士和全体纳税人，向为三明发展做出积极贡献的中央和省属驻明单位、驻明部队、武警官兵、公安干警，向关心支持三明发展的港澳台同胞、海外侨胞、国际友人，致以衷心感谢和崇高敬意！

　　我们清醒地看到，我市经济社会发展还存在不少困难和问题：区域经济竞争力不强，市区经济带动作用不突出，三明市区、沙县一体化及其与永安市城市联盟进展不快，城镇化水平不高；自主创新能力较弱，重点产业优势不突出，大企业、大项目不多，产业链延伸不够，产业集聚度不高；农业基础比较薄弱，农民持续增收难度加大；第三产业发展不够快，服务业配套设施建设滞后；部分经济指标增速趋缓，出口形势严峻，一些企业生产经营困难；三明沙县机场未能如期建成，市区四条通道建设进展不够快；就业保障、教育卫生、食品药品安全等关系群众切身利益的工作有待加强，节能减排压力较大,安全生产仍有隐患，影响社会稳定的因素不同程度存在；一些部门加快发展的紧迫感不强，观念不新，创新不够，办法不多，有的干部服务意识不强，作风散漫、推诿扯皮、办事拖拉、行政不廉的现象时有发生。对此，我们将高度重视，采取有力措施，认真加以解决。

　　二、2009年工作安排

　　今年是新中国成立60周年，也是推进“十一五”规划顺利实施的关键一年。面对新形势、新任务，困难和机遇并存，坚定信心，攻坚克难，促进增长，对于加快三明发展具有重要意义。我们要全面贯彻党的十七大、十七届三中全会和中央经济工作会议精神，以邓小平理论和“三个代表”重要思想为指导，深入贯彻落实科学发展观，按照省委八届五次全会、市委七届五次全会和省、市经济工作会议的部署，坚持“四求先行”、“四个重在”，着力民心、着力民生，进一步突出发展、注重运作、凝聚合力、弘扬正气，大力实施项目、品牌、创新、服务带动，加快落实“强化主轴、壮大两翼、‘块状’推进、连片发展”的区域发展思路和“突出工业、提升工业，加强农业、做特农业，培育三产、搞活三产”的产业发展思路，立足扩大内需、做强产业、改革开放、统筹协调、保障民生，促进经济持续较快增长，为推动三明在海西两个先行区建设中实现又好又快发展作出新的更大的贡献。

　　经济社会发展的主要预期目标是：全市生产总值增长12%；全社会固定资产投资增长25%；地方级财政一般预算收入增长12%；出口总值增长10%；按可比口径实际利用外商直接投资增长10%；社会消费品零售总额增长19%，居民消费价格总水平涨幅控制在4%左右；城镇居民人均可支配收入实际增长8%，农民人均纯收入实际增长6%；城镇登记失业率控制在4.2%以内；人口自然增长率控制在7.6‰以内；单位生产总值能耗降低4.2%，二氧化硫排放量削减率3%，化学需氧量排放量与上年持平。围绕上述目标要求，着重从以下四个方面开展工作：

　　（一）着力运作，力促增长。把促增长作为首要任务，继续组建项目团队，研究政策，抢占先机，强化运作，有效应对国际金融危机和宏观形势变化，进一步扩大内需，保持经济持续较快发展。

　　用足国家投资政策，促进基础设施加快建设。把握投资导向，做足、做大、做强、做优项目，着力实施150个市级重点项目，力争年度投资137亿元。特别是把基础设施建设作为拉动消费、促进增长的重中之重来抓，推进交通基础设施建设，完成泉三高速公路三明段扫尾工程，永武高速公路三明段基本完工，永宁高速公路实现全线开工，力争建泰高速公路控制性工程动工，深化三明绕城、三明至厦门、尤溪至莆田兴化湾、永安至漳州、三明至明溪等高速公路前期工作，实施9个国省干线网络公路改造建设项目。加快向莆铁路三明段建设；南三龙、浦建龙铁路今年立项，力争年底三明段控制性工程动工，确保2010年全线开工；深化长永泉铁路三明段前期工作；继续推进三明沙县机场建设；确保海西三明综合交通枢纽规划及三明国家公路运输枢纽“工可”获批，力争重点工程动工。加快电力项目开发，争取永安火电厂扩改项目1台机组投产，力争永安煤矸石综合利用项目获批，推进三明核电和大唐国际煤电项目取得突破性进展。确保500千伏超高压电网与南平、龙岩实现环网连接，开展第二座500千伏输变电工程前期工作，建设14座110千伏以上变电站，实施23个县域电网改造提升项目，力争新农村电气化建设走在全省前列。加强农业和林业基础设施建设，力争农田基本建设投资5亿元以上，土地整理10万亩以上，新建竹山道路1000公里，建成一批竹山灌溉设施。健全防灾减灾体系，加快海西三明气象中心建设，加强防汛抗旱和防台风工作，抓好以农村饮水安全、病险水库除险加固、灌区节水改造为重点的农村民生水利工程。管好用好建设资金，提高投资效益，建设精品工程。

　　用活适度宽松的货币政策，促进融资环境进一步优化。把握信贷规模宽松的政策，用好宽裕的流动性，积极上报信贷项目，争取信贷规模，力争新增贷款100亿元以上。加强金融服务，创新金融产品，简化贷款程序，调优信贷结构，着力支持重点产业、重点企业、重点项目，加大对“三农”、中小企业、个人消费、民生工程、节能减排、科技创新、技术改造和兼并重组、区域协调发展的信贷支持。突出政银企合作重点，实行产业转型升级重点项目银行贷款风险保证金制度，建立龙头企业资金链应急保障机制，鼓励和引导企业直接融资，稳步推进创业投资公司、村镇银行等新型融资组织的发展，积极争取筹建三明城区农村商业银行。按照政策性银行信贷导向，推进开发性金融合作，力争授信额度、新增合作贷款有较大突破。发挥中小企业信用担保机构风险补偿金的引导和促进作用，推广评、押、保、贷一体化担保贷款模式，加强金融机构与担保机构的合作，力争在担保金额、放大倍数上实现突破。创建信用乡村和企业，推进社会信用体系建设，防范和化解金融风险。

　　用好积极的财政政策，促进产业优化升级。落实增值税转型改革政策，实施20个高新技术产业项目、40个省级技改提升项目，新增10家国家级高新技术企业，争取50个以上项目列入国家、省科技计划盘子，抓好100项专利技术和科技成果转化，力争高新技术产业产值增长30%。落实产业优化升级和自主创新政策，争取“6·18”项目成果交易会签约项目数、总投资额、动工率继续走在全省前列，重点建设汽车及零部件、生物医药等公共研发平台，推动50%以上县（市、区）通过全国科技进步考核，提高技术创新能力。落实品牌带动扶持措施，新增2-4个中国驰名商标和中国名牌产品；新认证25个有机食品、绿色食品、无公害农产品，新增3-4个国家地理标志产品保护，建立5个农产品品牌基地；鼓励企业参与制定国家和行业标准，提升企业影响力。

　　（二）着力提升，优化结构。把转变发展方式和调整结构作为主攻方向，落实市委市政府《关于推进海峡西岸两个先行区建设实现三明又好又快发展的实施意见》，壮大重点产业，打造块状经济，推动连片发展，提升经济增长的质量和效益。

　　突出工业、提升工业。强化工业主导地位，优化工业结构，推动产业集聚，增强发展后劲，力争规模工业增加值增长19%。突出产业延伸，围绕壮大规模、连片发展，抓好投资千万元以上的21个冶金项目和31个机械项目，以主轴为重点打造冶金及压延、机械与汽车零部件产业区；实施投资千万元以上的60个林业项目、36个矿业项目、7个生物医药项目，以东南一翼、西北一翼为重点构筑林产工业、矿产品加工、生物医药产业区，力争“4+1”产业集群产值达到680亿元。同时，改造提升纺织、化工、建材等传统产业，实施一批重点项目，扩大规模效益。突出园区拓展，创新投入机制，完善基础设施，集聚关联企业，加快建设14个工业园区和专业园区，重点打造金属材料深加工、汽车及零部件、氟化工、铅锌矿深加工产业“板块”，培育一批特色园区，力争新增入园企业150家。提升质量效益，集中财政资金4500万元，支持工业增量提质；落实鼓励中小企业加快发展政策，对100家市场前景好、科技含量高的成长型中小企业进行贷款贴息；鼓励企业异地扩改，对退城入园企业实际缴纳的土地使用税由同级财政部分返还；加强生产要素配置，做好煤炭、电力、成品油供应和运力调度；引导有市场的企业快产多销，鼓励重点项目使用地材，帮助企业开拓市场，促进企业提速增效。提升发展能力，加快实施58个亿元以上工业项目，抓好136个工业新增长点项目；加大对重点企业的扶持力度，支持在明中央、省属企业新上项目、延伸产业，培育行业领军企业；加强与国内外大企业的合作，重点引进中央属企业投资三明，引入上市公司布局项目，实现借力发展。

　　加强农业、做特农业。突出产业化，扩大规模化，提升科技化，健全组织化，带动外向化，调整优化农业结构，加快发展现代农业。壮大农业优势产业，调动农民种粮积极性，鼓励发展粮食规模经营，扶持粮食大户、产粮大县发展，确保我市粮食生产总水平基本稳定。着力发展10个特色产业，重点建设10个现代农业示范园区、10个特色农业生产基地、10个特色农业示范乡镇，建立一批“一村一品”示范村和专业村。推进农业产业化经营，落实扶持农业产业化经营政策，重点扶持135家市级以上龙头企业，力争实现产值100亿元以上。建立农产品流通市场体系，扶持并新办一批运作规范、带动面较广的专业合作社，在全市建设6个批发市场，改造提升10个农村集贸市场和农产品专业市场。强化农业科技支撑，进一步深化“村会协作”，创新“村企协作”模式，完善“农业155”体系，实施农业“五新工程”，加强农村“六大员”队伍建设，培训农村实用人才6万人。推进农业标准化生产，强化重大动物疫病防控和农作物病虫害防治。推动农民持续增收，落实强农惠农政策，挖掘农业内部增收潜力，发展农村二、三产业，拓展农村劳动力就业渠道，引导返乡农民工积极创业，参与农业农村经济发展。

　　培育三产、搞活三产。把发展第三产业作为调整经济结构的“重头戏”，深入开展加快流通产业发展试点城市工作，壮大商贸物流，扩大消费需求。积极发展生产性服务业，加快推进海西金属材料制品市场、永安埔岭汽车商贸城等专业市场建设，开发建设三明城市物流园区、闽西北物流园区，抓紧实施三明“无水港”等项目；扶持一批骨干物流企业，壮大交通运输业；办好海峡两岸（三明）林业博览会、永安笋竹文化旅游节、沙县小吃旅游文化节等会展活动，发挥市科技会展中心的作用，培育会展经济。持续壮大消费性服务业，支持市商业、物资、医药等流通企业加快发展，积极发展信息服务业，鼓励发展科技服务、法律咨询等服务业，壮大物业管理、家政服务等社区服务业；建设无线宽带城市，扩大汽车、通信、文化、健身等热点消费；积极拓展农村消费，实施“家电下乡工程”，完善“万村千乡”市场工程，实现“农家店”覆盖全市所有乡镇和60%的建制村。坚持建筑业拓展和房地产业开发并举，有序推进房地产业发展。加快发展旅游业，打好旅游产业发展总体仗，强化泰宁世界地质公园龙头作用，围绕“休闲、安养、文化”主题开发重点旅游项目，培育精品旅游线路。完善旅游配套设施，抓紧建设一批四星级以上酒店。支持泰宁申报“中国丹霞地貌”世界自然遗产、创建5A级旅游景区，开展媒体营销、联合促销等活动，力争全年接待游客、旅游总收入分别增长20%以上。

　　推进区域联动发展。围绕主体功能区规划，强化主轴，抓好联动项目，加快城市规划、交通通道、产业布局、项目运作一体化进程，逐步形成城市联盟。壮大两翼，引导东南一翼构建沿海产业转移集中区、优势资源深度加工区、品牌农业集中区，推动西北一翼发展主业凸显、产业集聚的特色经济示范区，力争地区生产总值、财政收入增幅超过全市平均水平，增强县域经济实力。加快城镇建设，完善县城设施，促进产业集聚，构建各具特色的城区；继续推进5个县域次中心建设，打造25个特色小城镇；突出规划先行、典型引路、完善设施、统筹协调，加快推进新农村建设，年内建设30个示范村、200个推进村。支持老区发展，从项目、资金等8个方面落实“老区优先、适当倾斜”政策，发挥中央苏区、老区优势，争取国家、省里更大支持。推进扶贫开发，着重抓好38个省级、85个市级扶贫村建设，实施少数民族乡村帮扶工程，继续做好库区移民安置和后期扶持工作。

　　强化市区经济的带动作用。突出重点产业，完善园区共建机制，推进梅列经济开发区、三元经济开发区、金沙园、埔岭汽车工业集中区建设，支持厦工三重、双轮化机、三明机床等企业加速拓展，实施30万吨二甲醚等重点项目，壮大市区工业和商贸物流业。突出重要板块，徐碧新城重点推进中央商务区、五星级大酒店等项目建设，下洋中区抓紧实施水产品批发市场、白沙片区改造等项目；台江新区建成台江大桥，并推动新区基础设施等项目建设。突出通道建设，加快贵溪洋大道、徐碧大桥至陈大及翁墩立交、工业南路至泉三高速公路南互通通道建设，尽早动工富兴堡至三元区委党校通道，拓展市区空间。

　　（三）着力先行，增强活力。以改革开放为动力，着力于“求”，着眼于“先”，争创区域特色，增强发展活力，进一步提升竞争力。

　　在深化改革上先行。推进农村综合改革，稳定和完善农村基本经营制度，创新农业经营方式，建立农业投入保障机制。规范农村土地管理，坚持最严格的耕地保护制度，实行最严格的节约用地制度，确保耕地占补平衡。依法、自愿、有偿流转土地承包经营权，发展适度规模经营。推进乡镇机构改革，开展小城镇机构改革试点工作，构建农村公共服务平台。深化集体林权制度改革，巩固扩大林改成果，推进商品林采伐制度等配套改革，健全完善林业管理体系，拓展林权抵押贷款，集体林权制度改革继续走在全国前列。建立健全现代企业制度，支持和推动企业上市，力争新增2家以上上市企业。落实国有资产出资人和国有产权代表制度，支持国投公司参与资源综合利用、重点行业等领域的开发与发展。应对财税体制改革新变化，加快培育新的财税增长点，促进财税收入稳步增长。优化财政支出结构，深化部门预算改革，在市级所有预算单位实行国库集中支付改革，加强行政事业单位资产管理，基本完成农村义务教育债务清理化解工作。严肃财经纪律，加强财政监督和审计监督。稳步实施政府机构改革，推进事业单位分类改革。

　　在扩大开放上先行。强化明台合作，主动承接台湾产业转移，加快海峡两岸（三明）现代林业合作实验区建设，推进清流台湾农民创业园和台商投资集中区开发，抓好10项明台合作项目，带动两岸文化、教育、科技、旅游等领域交流。拓展对外经贸，用好“9·8”等招商平台，开展境外专场招商，继续组织重点招商、小分队招商和产业定向招商，力争签约合同项目批办率达到50%以上。落实出口扶持政策，重点抓好一批生产型自营出口企业和流通型外贸出口企业，鼓励企业进口资源性商品和国外先进装备、技术，支持优势企业参与境外资源开发和技术合作。加强海外华人华侨联络工作，密切与港澳地区、海外华侨及国外友城的沟通联系。深化区域协作，建设大田京口沿海产业转移项目区、尤溪临港建材工业集中区，开发宁化、建宁等省际边界产业集中区和物流园区，争取全年引进区外资金增长30%。

　　在提升非公有制经济上先行。落实扶持政策，注重搞好服务，推进“小巨人”企业加快发展，新增5家产值超亿元的非公企业，新增20家纳税百万元以上非公企业。支持项目建设，搞活商会经济，激活民间资本，引导三明籍在外人士回乡创业，鼓励开发重大项目，新增非公企业投资项目160个。发展总部经济，支持国内外知名企业在三明设立具有法人资格的生产、营销机构，鼓励总部外迁企业回归，力争全年新增总部经济企业3家。

　　（四）着力统筹，改善民生。把以人为本作为出发点和落脚点，注重统筹兼顾，推进经济社会协调发展，促进人与自然可持续发展，保持社会和谐稳定。

　　加强节能减排和生态建设。强化节能降耗，继续实施节能“十大工程”，推进50个重点节能改造项目，抓好11家省级循环经济工业示范企业（园区），淘汰落后水泥产能，确保30家企业通过清洁生产审核验收。推进污染减排，启动三明垃圾焚烧发电厂项目，建成将乐、尤溪、大田、宁化污水处理厂和泰宁县垃圾无害化处理场，开工建设明溪、清流、建宁污水处理厂和永安污水处理厂二期项目。持续开展规模化畜禽养殖污染治理，继续推进闽江流域三明辖区水环境治理，抓好重点企业烟气脱硫和“上大压小”项目。加强生态建设，完善生态补偿机制，加强生态环境功能区划和建设；强化东牙溪饮用水源保护，启动市区第二水源建设，完成乡镇饮用水源保护区规划编制工作，保障群众饮水安全。

　　加快发展社会事业。加强素质教育，推动义务教育均衡发展，做优做强优质高中，加强对教师特别是农村教师的培训；三明学院力争通过教育部本科教学工作水平评估，提升职业技术学院教学质量和科研水平；大力发展职业教育，积极推进中等职业教育资源整合；重视学前教育，关心特殊教育，发展远程教育和继续教育，支持、引导民办教育，关心外出和外来务工人员子女的学习、生活。围绕重才、育才、聚才、用才，引进高层次和紧缺急需专业人才，健全激励和保障机制，完善人才公共服务平台，发挥各类人才的作用。健全公共卫生体系，推进医药卫生体制改革，加强重大疾病防控工作，完善卫生监督体系，推进农村和城市社区卫生服务体系建设，发展中医药和妇幼保健事业，扶持5个特色诊疗中心建设。繁荣文化事业，发展文化产业，争创一批全国重点文物保护单位和国家级历史文化名村，抓好中村回瑶文物园区等项目的保护与开发，加强网络文化建设和管理。开展全民健身运动，发展竞技体育，争取实现全运会夺金目标。稳定低生育水平，健全人口和计划生育工作长效机制，加强流动人口管理服务，继续抓好出生性别比失调的治理，力争新增国家级和省级计生优质服务先进县各1个。加快发展社会科学，繁荣新闻出版、广播电视、文学艺术。深入实施全民科学素质行动计划纲要，提升全民科学素质。落实老干部和老年人优待政策，发展老龄事业。继续加强统计工作，完成第二次全国经济普查。促进民族宗教、档案、史志、水文、防震等工作全面进步。

　　促进社会和谐稳定。密切与各民主党派、工商联、无党派人士的联系与协商，发挥工会、共青团、妇联等人民团体的桥梁纽带作用。完善村（居）民自治，依法有序开展基层自治组织换届选举。持续开展普法教育，加强法律服务和援助工作。推进社会主义核心价值体系与和谐文化建设，以“共建共享和谐三明”为目标，深入开展群众性精神文明创建活动，强化城市长效管理，巩固、发展创建全国文明城市工作先进城市、国家卫生城市和国家园林城市成果。落实社会治安综合治理领导责任制，开展新一轮争创全国社会治安综合治理优秀地市工作，健全社会治安防控体系，创新城乡社区警务模式，依法严厉打击和预防各种刑事犯罪，增强群众安全感。重视未成年人思想道德教育，预防青少年违法犯罪。加强信访工作，完善化解矛盾纠纷长效工作机制，重视解决信访突出问题，预防和有效处置群体性事件。落实安全生产责任制，强化重点行业和重点领域的安全监管，防范重特大事故发生。健全应急管理体制机制，提高处置突发事件的能力。健全价格监测预警和应急机制，保持物价基本稳定。加强民兵预备役建设，做好国防动员、人民防空、双拥共建工作，巩固军政军民团结。

　　加强就业和社会保障。实施更加积极的就业政策，深化自主创业活动，有针对性地开展劳动就业培训，加快培养各类技能人才，新增城镇就业人员2.2万人。完善劳动关系三方协调机制，努力构建和谐劳动关系。扩大养老、失业、工伤等社会保险覆盖面，健全城镇职工基本医保、城镇居民基本医保、城乡医疗救助制度，继续提高企业退休人员养老金标准和优抚对象生活补助标准，增加城市和农村低保补助，探索建立被征地农民就业和社会保障制度。维护妇女儿童合法权益，大力发展慈善事业，支持社会救助活动。

　　抓好为民办实事和惠民项目。城乡教育方面，继续实施农村义务教育阶段寄宿生宿舍建设工程；对城乡低保家庭高中学生实行助学金；对中职学校涉农专业学生及城市低保家庭学生减免学费。医疗卫生和人口计生方面，改造提升一批县级医疗机构和乡镇卫生院，推进社区卫生服务中心规范化建设，完善新型农村合作医疗和补充医疗保险制度；继续开展“光明行动”；实施残疾人康复工程；新建、改扩建5个县级计划生育服务站；提高农村部分计划生育家庭奖励扶助标准。文化建设方面，新建、改扩建15个乡镇综合文化站；加快有线电视数字化整体转换；推进“农家书屋”建设；建设群众体育健身工程。城市公共设施方面，建设市老年活动中心、老年公寓和社会福利中心，实施下洋体育健身中心项目，改造社区基础设施；建设一批停车场和公交停靠站，创建公交精品示范线路；开展引进液化天然气项目前期工作。就业和社会保障方面，完成农村劳动力转移培训4.1万人，实现转移就业4.5万人；做好大学生参加城镇居民基本医疗保险工作；完善政策性农业保险试点工作，实施自然灾害救济救助公众责任保险；新建和改扩建15所农村敬老院；继续抓好廉租住房、经济适用住房、限价商品房建设。强农惠农方面，继续发展农村户用沼气；深入开展农村家园清洁行动；实施农村公路网络工程；推进农村危房改造工程；实现“造福工程”年度搬迁1万人；对村计生协会会长、妇代会主任、团支部书记发放津贴，提高村主干和边远建制村乡村医生的补贴补助标准。援建灾区方面，实施川西旅游环线道路改造等7个援建彭州市红岩镇项目。食品药品安全方面，深化产品质量和食品药品安全专项整治，继续治理“餐桌污染”，建设“食品放心工程”；建立农产品质量安全检验检测体系。和谐平安方面，巩固深化平安创建活动；继续帮助驻明部队建设。

　　三、切实加强政府自身建设

　　突出求真务实，建设开明政府。决策科学民主，落实重大事项决策程序规定，建立健全公众参与、专家论证和政府决定相结合的行政决策机制，实行决策责任追究制度，做到政府的决策和工作，经得起实践的检验、人民的检验、历史的检验。承诺求实诚信，讲实话、办实事、求实效，对每年确定的重点工作、重要项目和民生事项，都进行责任分解，向人大代表、政协委员作出承诺，并在第二年“两会”上报告落实情况，把说过的话兑现好，发过的文落实好，承诺过的事解决好，提高政府公信力。运行有序透明，拓展政务公开，实行政府信息全面公开，加强电子政务和政府网站建设，建立民生资讯服务平台，保障群众对政府工作的知情权、参与权、表达权、监督权。

　　突出提高效率，建设服务政府。提高创新力，开展深入学习实践科学发展观活动，解放思想、勇于创新，敢于打破常规，少说“不能做”，多想“怎么做”。提高执行力，善于把握机遇，积极争取项目获批、政策支持、资金扶持，集中力量突破重点工作，加大力度破解发展难题，深入基层解决问题、推动落实。提高行政效能，按照国家部署取消和停止100项行政事业性收费，继续精简审批事项，下放经济社会管理权限，扩大网上审批，推行项目联审联办，做到特事特办、特事快办。加强公务员队伍建设，抓好绩效管理，精简会议、文件，按照“四个不让”的要求，开展“比服务、比效率、比质量、比作风、比形象”的竞赛活动，强化机关效能督查，查处损害发展环境的行为。

　　突出依法依规，建设法治政府。认真执行《监督法》，自觉接受人大及其常委会的法律监督、工作监督，向人大及其常委会报告工作，执行人大及其常委会决议；接受政协的民主监督，向政协通报情况，办好人大代表建议和政协提案。坚持依法行政，贯彻落实《国务院关于加强市县政府依法行政的决定》，把依法行政贯穿到政府决策、执行、督查的全过程，提高依法行政能力。严格行政执法，完善行政执法经费保障机制，规范行政处罚自由裁量权，强化行政执法责任追究，提高行政执法效率和水平。畅通行政复议渠道，有效化解行政争议。

　　突出源头治理，建设廉洁政府。从筑牢思想防线抓起，倡导“八个方面”良好风气，讲党性、重品行、作表率，克服官僚主义、形式主义，解决作风散漫、行政不廉等问题。从落实制度抓起，严格执行党风廉政建设责任制，建立健全惩治和预防腐败体系，落实廉洁自律各项规定，规范权力运行，加强资金监控，查办违法违纪案件。从加强薄弱环节抓起，深化纠风专项整治，坚决防止和纠正漠视群众疾苦、与民争利的行为，树立清正、廉洁、为民的形象。

　　各位代表！海西应先行，三明要争先。让我们紧密团结在以胡锦涛同志为总书记的党中央周围，高举中国特色社会主义伟大旗帜，以邓小平理论和“三个代表”重要思想为指导，深入贯彻落实科学发展观，在市委的正确领导下，团结和带领全市人民，以只争朝夕的精神，坚韧不拔的毅力，改革创新的锐气，埋头苦干，奋勇前进，为推进两个先行区建设，实现三明又好又快发展而努力奋斗！
